# Regression Analysis of Protoporphyrin IX Measurements Obtained during Dermatological Photodynamic Therapy

**DOI:** 10.3390/cancers11010072

**Published:** 2019-01-10

**Authors:** Jessica Tyrrell, Cheryl Paterson, Alison Curnow

**Affiliations:** European Centre for Environment and Human Health, University of Exeter Medical School, University of Exeter, Knowledge Spa, Royal Cornwall Hospital, Truro, Cornwall TR1 3HD, UK; J.Tyrrell@exeter.ac.uk (J.T.); C.B.Paterson@exeter.ac.uk (C.P.)

**Keywords:** aminolevulinic acid (ALA; Ameluz), dermatology, fluorescence, imaging, methyl aminolevulinate (MAL; Metvix), non-melanoma skin cancer (NMSC), photobleaching, photodynamic therapy (PDT), protoporphyrin IX (PpIX)

## Abstract

Photodynamic therapy (PDT) is a light activated drug therapy that can be used to treat a number of dermatological cancers and precancers. Improvement of efficacy is required to widen its application. Clinical protoporphyrin IX (PpIX) fluorescence data were obtained using a pre-validated, non-invasive imaging system during routine methyl aminolevulinate (MAL)-PDT treatment of 172 patients with licensed dermatological indications (37.2% actinic keratosis, 27.3% superficial basal cell carcinoma and 35.5% Bowen’s disease). Linear and logistic regressions were employed to model any relationships between variables that may have affected PpIX accumulation and/or PpIX photobleaching during irradiation and thus clinical outcome at three months. Patient age was found to be associated with lower PpIX accumulation/photobleaching, however only a reduction in PpIX photobleaching appeared to consistently adversely affect treatment efficacy. Clinical clearance was reduced in lesions located on the limbs, hands and feet with lower PpIX accumulation and subsequent photobleaching adversely affecting the outcome achieved. If air cooling pain relief was employed during light irradiation, PpIX photobleaching was lower and this resulted in an approximate three-fold reduction in the likelihood of achieving clinical clearance. PpIX photobleaching during the first treatment was concluded to be an excellent predictor of clinical outcome across all lesion types.

## 1. Introduction

Photodynamic therapy (PDT) is a light activated drug therapy that can be used topically to treat a number of non-melanoma skin cancers (NMSC) and precancers [1]. NMSC management commonly includes surgical excision, 5-fluorouracil or cryotherapy [2]. These therapies are not always associated with excellent cosmesis and their appropriateness can be limited, depending on the location, size and number of lesions to be treated [2,3,4]. PDT uses light to activate a pre-administered drug in the presence of molecular oxygen to kill diseased cells without harming surrounding connective tissue, so that healing tends to occur without scarring [1]. It also has several advantages, including the ability to treat a whole area of field change, good lower leg healing, repeated treatment without patient resistance and excellent cosmesis in highly visible sites without advanced surgical techniques [3,4,5]. PDT can also be utilized as a treatment adjuvant [5]. Dermatological PDT has been found to be safe (with few side effects beyond treatment effects) and efficacious for the treatment of actinic keratosis (AK), superficial basal cell carcinoma (sBCC) and Bowen’s disease (BD) [3,4,5,6].

Protoporphyrin IX (PpIX) is the photosensitizer most commonly used in dermatological PDT [3]. A topical cream containing a small, soluble precursor to PpIX (e.g., 5-aminolevulinic acid (ALA) or its methyl ester, methyl-aminolevulinate (MAL)) is utilized because PpIX is relatively large and water-insoluble [1]. PpIX precursors are absorbed and enzymatically converted into PpIX over three hours by the haem biosynthesis pathway naturally present in all nucleated cells [7]. Neoplastic cells accumulate more PpIX more rapidly than normal cells because their haem biosynthesis is elevated/less well controlled, and this creates a relatively selective treatment window in which 635 nm irradiation can be applied to activate PpIX [1]. The disrupted tumor surface is also more permeable than healthy skin to the topically applied prodrug, facilitating PpIX precursor penetration [1].

PpIX also exhibits characteristic red fluorescence (at 635 nm and 700 nm) when excited by blue light (410 nm) and therefore cells accumulating PpIX can be identified through fluorescence monitoring [8,9]. Photodynamic detection (PDD) of this fluorescence can aid the identification of pre-clinical lesions in an area of field change or the margin of a poorly demarcated lesion and thus ensure that all the skin disease is properly eliminated during excision [10,11]. However, PpIX fluorescence can also now be exploited to follow the changes in PpIX concentration within the skin during PDT. Our ability to do this has been limited in the past by the poor reproducibility of results and numerous factors influencing fluorescence detection [12,13] and so historically, invasive techniques (such as chemical extraction) have been commonly utilized to determine the presence and concentration of tissue PpIX [14,15]. We therefore developed and validated [16], a non-invasive imaging system based on a commercially available piece of PDD equipment (Dyaderm, Biocam, Regensburg, Germany) [17] in order to monitor PpIX fluorescence changes in real-time during routine dermatological MAL-PDT of licensed skin lesions [18]. We found that both PpIX accumulation and photobleaching are important indicators of dermatological MAL-PDT treatment success and anything that adversely affected them had the potential to reduce treatment efficacy. 

## 2. Results

### 2.1. Demographics

Initial analyses checked whether there were any demographic differences between the participants with complete PpIX fluorescence data ± outcome data. Table 1 indicates that no significant differences were detected for any of the demographic variables considered. Clinical outcome data was available for the majority (n = 172; 83.1%) of participants with complete fluorescence data sets, with complete clinical responses being observed in 75.6% (n = 130) of these participants at three months. Partial responses were recorded in 23.8% (n = 41) of cases and no response in one single case only (0.6%; n = 1). Mean participant age was 73.1 years, with a roughly equally gender distribution (47.1% male). Lesion type distribution was also approximately equal, with 37.2% AK, 27.3% sBCC and 35.5% BD. The majority of lesions were located on the head (39.5%), body (22.7%) and legs (24.4%), with the remainder either being acral (8.7%) or on the arms (4.7%). A considerable number of participants (n = 77; 44.8%) also utilized some form of pain relief, with the majority (93.5%) using an air cooling device (ACD).

### 2.2. Different Lesion Types

**Figure 1:**
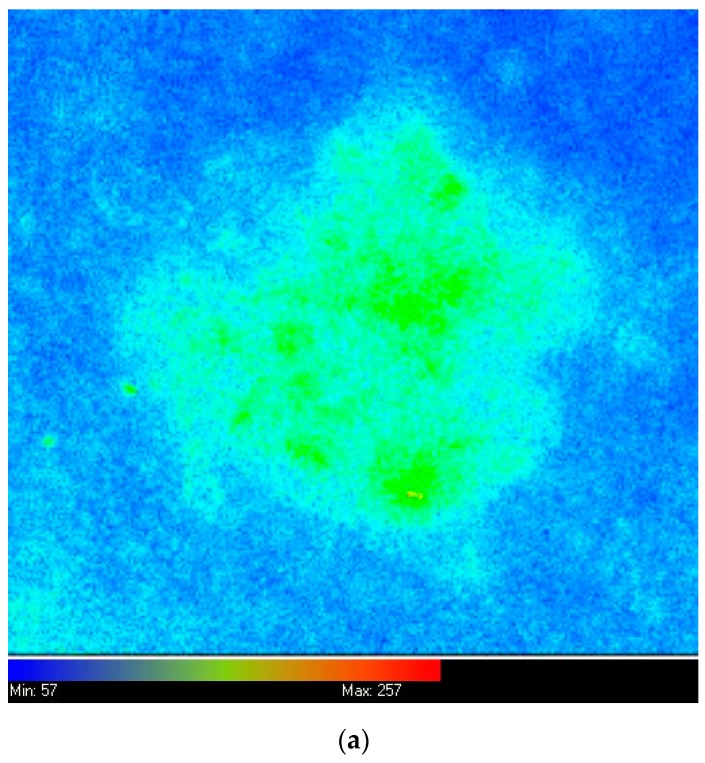

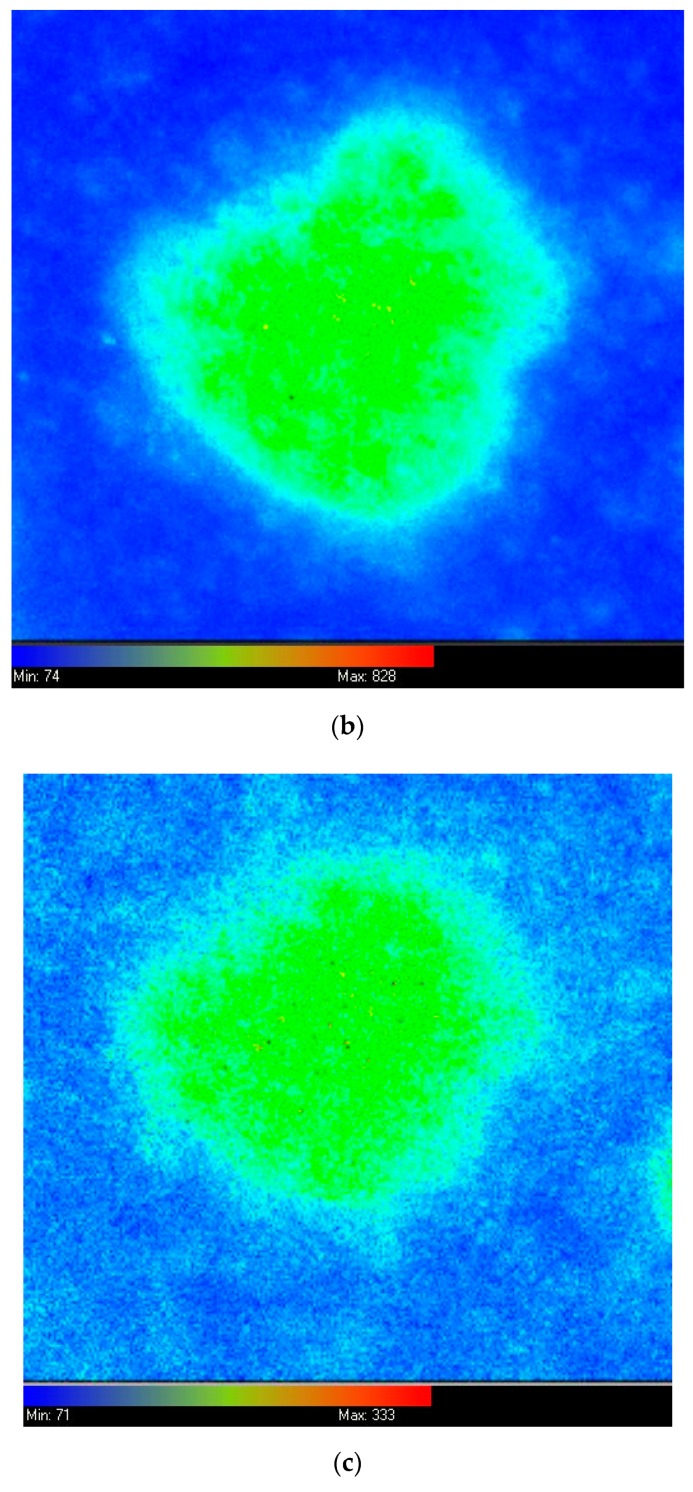

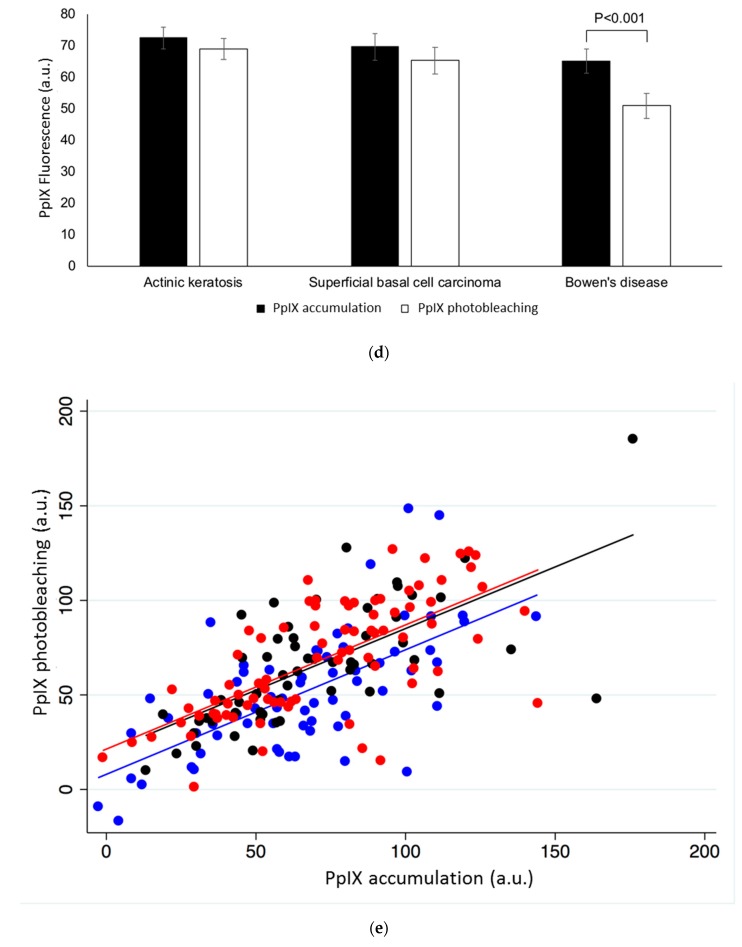
Representative (false color coded) PpIX fluorescence images of an individual superficial basal cell carcinoma (sBCC) (upper back, 70 year old female) (**a**) before methyl aminolevulinate (MAL) application, (**b**) three hours later prior to irradiation and (**c**) immediately following irradiation. Actinic keratosis (AK), sBCC and Bowen’s disease (BD) lesions (**d**) accumulate more PpIX than is photobleached during MAL-induced PDT irradiation (mean PpIX fluorescence is presented in arbitrary units (a.u.) with bars indicating the standard deviation) and (**e**) PpIX accumulation and photobleaching (a.u.) is correlated in each licensed lesion type during MAL-PDT, with red dots representing AK, black dots representing sBCC and blue dots representing BD. The lines of best fit are also presented and the R^2^ values for each lesion are: AK 0.47, sBCC 0.45 and BD 0.43; overall R^2^ = 0.45).

Three PpIX fluorescence images were taken of every skin lesion, one before MAL application, one after MAL application and one immediately following the irradiation period. Representative images from a single lesion are presented in Figure 1a–c respectively. When mean PpIX accumulation and photobleaching was considered from all of these images combined at each of these time points, the former was found to be higher than the latter in all three lesion types investigated, with the strongest evidence (*p* < 0.001) in BD lesions (Figure 1d). This confirmed the trends observed in our previous work [19] and suggested that accumulation is a prerequisite for photobleaching with higher PpIX levels prior to irradiation, resulting in greater photobleaching during irradiation. PpIX accumulation and photobleaching were clearly correlated in all three lesion types (R^2^ for each individual lesion type: AK 0.47, sBCC 0.45 and BD 0.43; overall R^2^ = 0.45; Figure 1e). 

**Figure 2:**
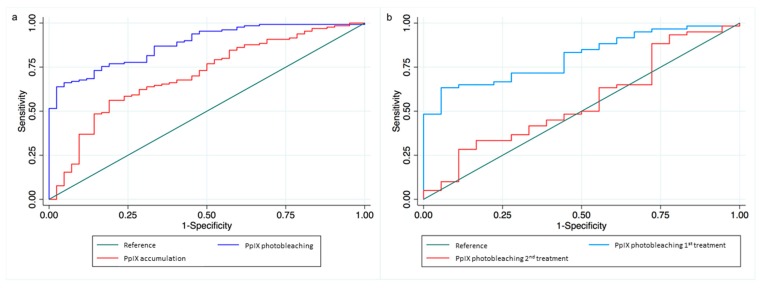
(**a**) Receiver operating characteristic (ROC) curve shows that PpIX photobleaching predicts clinical outcome at 3 months more successfully than PpIX accumulation. Area under curve (AUC) is 0.88 for PpIX photobleaching and 0.69 for PpIX accumulation and (**b**) ROC curve for first and second treatment PpIX photobleaching in the patients undergoing two treatments 9 days apart. AUC indicates first treatment PpIX photobleaching is a better predictor of clinical outcome (0.81 versus 0.55).

Treatment outcome at three months was found to be strongly associated with PpIX photobleaching during the first PDT treatment. When all licensed lesions were considered together, participants in the highest 50% of PpIX photobleaching were found to be 40 times more likely to achieve a complete clearance than those in the bottom 50% Odds Ratio (OR): 40.3 (95% Confidence Intervals (95%CI): 9.2, 176.3; *p* < 0.001). A 20% higher PpIX photobleaching was associated with nearly four-fold higher odds of complete clinical clearance (OR: 3.9 95%CI: 2.5, 6.0; *p* < 0.001). This was consistent across all lesion types (AK OR: 3.8 (1.8, 8.3; *p* < 0.01); sBCC OR: 5.0 (1.7, 15.4; *p* < 0.01); BD OR: 3.9 (1.9, 8.0; *p* < 0.001)) and indicated that lower PpIX photobleaching appeared to negatively affect efficacy independently of lesion type. When PpIX accumulation was considered, it was observed to be associated with treatment outcome to a lesser extent than PpIX photobleaching when all lesion types were combined. Individuals accumulating the highest 50% of PpIX were at 4.0 higher odds of complete clinical clearance (95%CI: 1.8, 8.7; *p* < 0.01) than those in the lowest 50%. A 20% higher accumulation was associated 1.7 higher odds of complete clinical clearance (95%CI: 1.3, 2.2; *p* < 0.001). Receiver Operating Characteristic (ROC) curve analyses highlighted the potential of PpIX photobleaching as a predictor of clinical outcome, with an area under the curve of 0.9, in comparison to 0.7 for PpIX accumulation (pdifference < 0.001; Figure 2a). This was similar for all lesion types with area under the ROC of 0.9, 0.9 and 0.9 for PpIX photobleaching compared to 0.7, 0.6 and 0.8 for PpIX accumulation in AK, sBCC and BD respectively. 

Half of study participants (*n* = 86) received two PDT treatments (27 AK, 26 sBCC and 33 BD). As previously observed [19], both PpIX accumulation and photobleaching were consistently significantly reduced on the second PDT treatment in all three lesion types (mean PpIX accumulation and photobleaching for all lesion types combined on the first treatment here =70.0 a.u. and 63.0 a.u. versus 46.2 a.u. and 46.5 a.u. on the second treatment, *p* < 0.001 and *p* < 0.001 respectively). Neither the PpIX accumulation nor photobleaching observed during the second PDT treatment was associated with clinical outcome. However, when PpIX photobleaching across both treatments were combined, a 20% higher overall PpIX photobleaching was associated with 1.7 higher odds of complete clinical clearance (95%CI: 1.1, 2.5; *p* < 0.05). PpIX photobleaching during the first treatment was a better predictor of clinical outcome than that during the second treatment in the individuals undergoing two treatments (area under ROC 0.8 and 0.6 respectively; pdifference < 0.01; Figure 2b).

### 2.3. Age and Gender

**Figure 3:**
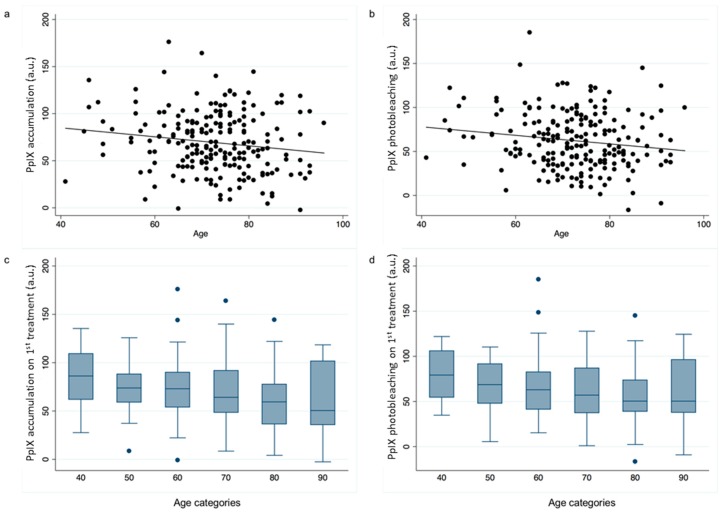
Scatter plots of age at treatment versus (**a**) PpIX accumulation (a.u.) and (**b**) PpIX photobleaching (a.u.) and box plots of (**c**) PpIX accumulation and (**d**) PpIX photobleaching distribution for different age categories.

Participant age was found to be inversely associated with both PpIX accumulation and subsequent photobleaching when all these data were analyzed as a whole (Figure 3). A one-year increase in age was associated with both a 0.5 unit lower PpIX accumulation (95%CI: 0.1, 0.9; *p* < 0.05) and a 0.5 unit lower PpIX photobleaching (95%CI: 0.1, 1.0; *p* < 0.05). However, at the individual lesion type level these associations were not observed in AK or BD lesions, only in sBCC where for every one-year increase in age a 1.1 unit decrease in PpIX accumulation alone (95%CI: 0.2, 2.0; *p* < 0.05) was observed. Participant age was not found to be associated with the clinical outcome at three months (OR: 1.0 (95%CI: 1.0, 1.0); *p* = 0.54). 

Participant gender was found to neither alter PpIX accumulation (*p* = 0.53) nor photobleaching (*p* = 0.29) when all the lesion types were combined or effect the subsequent treatment outcome (*p* = 0.78).

### 2.4. Lesion Location

**Figure 4:**
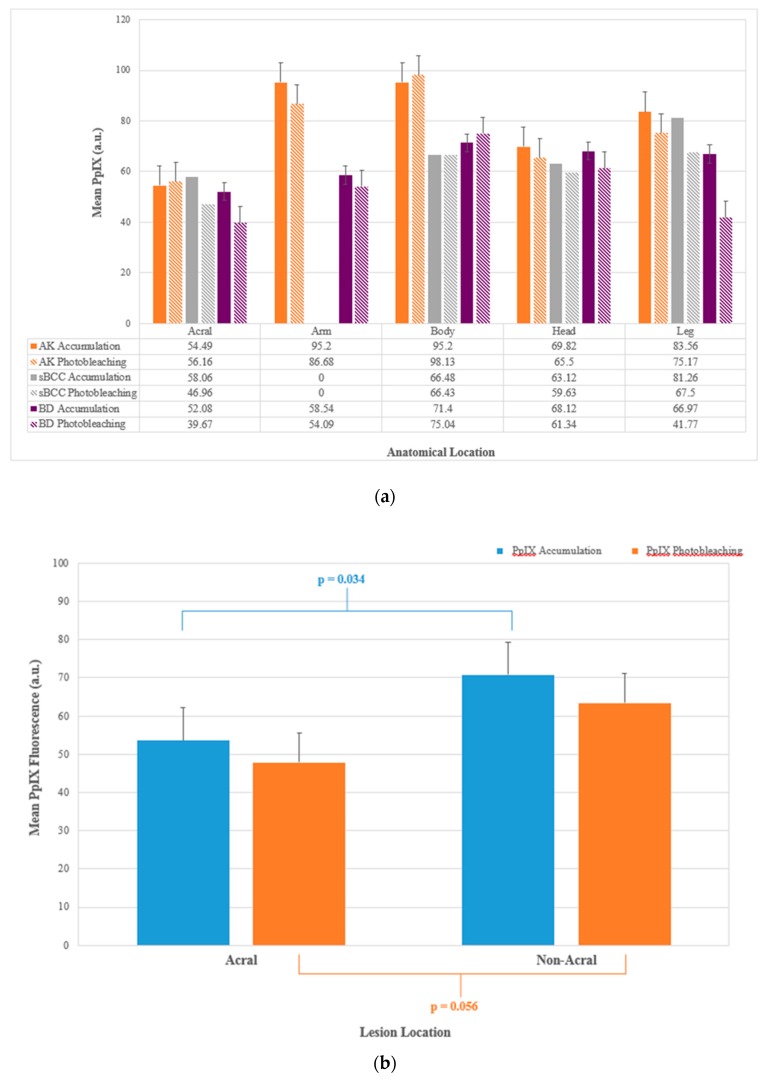
(**a**) Mean PpIX accumulation (a.u.) and mean PpIX photobleaching (a.u.) recorded during MAL-PDT conducted in different licensed indications (AK, sBCC and BD) on different anatomical locations and (**b**) comparison of mean PpIX accumulation and mean PpIX photobleaching in acral versus non-acral lesions (AK, sBCC and BD) undergoing MAL-PDT. Bars on both panels represent the standard deviations of the data.

Only 17 lesions (8.2%; 8 AK, 1 sBCC and 8 BD) in the whole data set were classified as acral (fingers and toes). Individuals with acral lesions were generally found to be of a similar age and sex distribution to those with non-acral lesions. PpIX accumulation was observed to be lower in acral lesions and PpIX photobleaching was approaching significance (*p* = 0.034 and 0.056 respectively) within this small data subset (Figure 4) supporting our previous observations in this respect [20]. 

As acral lesion numbers were limited, lesions located on the arms and legs were combined with these data and reclassified as “peripheral” for subsequent analyses. This again indicated a reduction in treatment efficacy at three months (OR: 0.5 (0.2, 0.9; *p* < 0.05), with lesions located on the head and main body being more than twice as likely to achieve complete clearance than peripheral lesions located on the limbs.

### 2.5. ACD Pain Relief

**Figure 5:**
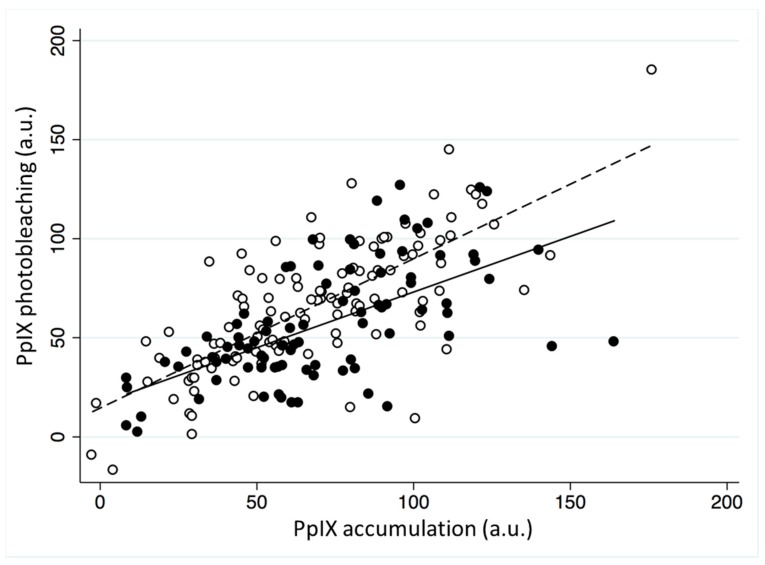
The effect of using air cooling pain relief on the correlation between mean PpIX accumulation (a.u.) and mean PpIX photobleaching (a.u.) during MAL-PDT of AK, sBCC and BD. Black dots represent the pain relief users, with the solid black line representing the line of best fit for pain relief users and the white dots represent no pain relief, with the dashed line representing the line of best fit for no pain relief. The regression coefficients from these models differ (*p* < 0.05).

Seventy-seven participants (44.8%) utilized some form of pain relief, with the majority (93.5%) using ACD. Analysis indicated that there was no association between PpIX accumulation and the use of ACD (*p* = 0.52), however PpIX photobleaching was lower (*p* < 0.05). This difference did not persist when analyzed on a lesion specific basis. The use of pain relief resulted in less photobleaching of the accumulated PpIX (Figure 5) and this was also noted in sBCC alone. 

The use of ACD was also associated with lower odds of achieving a complete clinical clearance at three months (OR: 0.4 (0.2, 0.7; *p* < 0.01), the equivalent to an approximate three-fold reduction. Adjustments for age and sex did not alter the likelihood of achieving clinical clearance but when this relationship between ACD usage and clinical outcome was considered by lesion type, BD was particularly affected and AK unaffected (AK OR: 0.8 (0.2, 2.9) *p* = 0.76); sBCC OR: 0.3 (0.1, 1.3) *p* = 0.11; BD OR: 0.2 (0.1, 0.6) *p* = 0.004). Participants who utilized ACD pain relief during both PDT treatments were noted to be even less likely to achieve a complete clearance (OR: 0.2 (0.0, 0.8)).

## 3. Discussion

The real-time PpIX fluorescence monitoring presented here and previously [19], clearly indicates that sufficient PpIX accumulation occurs during routine dermatological MAL-PDT of AK, sBCC and BD, when utilizing the well documented licensed MAL-PDT protocol derived for this purpose [3,18]. PpIX photobleaching during the first PDT treatment was observed to be strongly associated (*p* < 0.001) with clinical outcome at three months, further supporting our initial findings [21]. With further study, monitoring this variable may enable treatment success to be determined at the end of the first irradiation period and thus how many repeat treatments an individual patient/lesion may need to receive (for patient benefit and cost efficiency). Alternatively, this mechanistic insight may help us derive new PpIX-PDT protocols for other dermatological applications. It can be postulated that the results between the first and second PDT treatments may have been found to be significantly different, at least in part, because the amount of disease being treated in the second treatment was much reduced due to the effectiveness of the initial PDT treatment. 

As PpIX accumulation has been observed to be a prerequisite for PpIX photobleaching and the latter is now observed to be correlated with PpIX-PDT efficacy, anything that reduces either of these two variables has the potential to reduce treatment effectiveness. This was observed to some extent with the decline in PpIX accumulation and thus photobleaching with increasing patient age, fortunately this reduction was not substantial enough to adversely affect treatment outcome. However, the reduced PpIX accumulation in acral lesions indicated that a modified protocol may need development for this subset of difficult to treat lesions. A recent randomized controlled trial of acral AK by Nissen et al. [22], found that conducting MAL-PDT in association with curettage improved treatment outcomes as did increasing the drug-light interval to 21 h, however they concluded that there was an optimal amount and localization of PpIX required for best effect with minimal side effects. Interestingly here, even lesions on the limbs were less than half as likely to completely respond to MAL-PDT when compared with lesions located on the head and main body and brings new insight to our previous work [20]. Furthermore, skin is known to be very different at different points of the human body, varying greatly in thickness (e.g., thin on the face versus thick on the palms) and in specialization (number of blood vessels, hair follicles, sebaceous glands etc.). Any one of these parameters might influence PDT, particularly in terms of prodrug cream penetration, and thus may also effect treatment efficacy. Although not investigated here, poor technique when conducting MAL-PDT (e.g., poor lesion preparation) could also adversely affect clinical outcomes. It may also be possible in the future to improve outcomes in patients who accumulate high PpIX levels prior to irradiation but then experience relatively low photobleaching during irradiation, by extending the light delivery period to increase the light dose applied if substantial PpIX levels remain at the end of the standard light delivery period.

The biggest threat to MAL-PDT treatment success detected, was the use of air cooling. A significant reduction (*p* < 0.05) in PpIX photobleaching was observed in the combined licensed lesions and the odds of achieving a complete clinical response dropped to only 0.4. This latest evidence corroborates and extends the findings of our initial analyses in this respect [23], which noted that the ACD system employed in our Dermatology clinic (SmartCool, Cynosure UK Ltd., Cookham, UK) produced air at −35 °C locally directed via a hand-held nozzle. The application of this system to an area of normal skin in a healthy volunteer [23] resulted in significant cooling of the area from 30.3 ± 0.3 °C to 4.1 ± 0.4 °C over the course of eight minutes with a corresponding 16% decrease in oxygen saturation indicating that vasoconstriction occurred [23]. The high usage of ACD in this non-interventional observational study (93.5% of 44.8% pain relief users), may have also contributed to the relatively low complete clinical response rates observed (75.6%). Much higher treatment efficacy has been reliably documented for dermatological MAL-PDT [3,17] and as a result our practice has been altered. An alternative approach would be to reduce the fluence rate of the light delivery, delivering the total light dose over an extended period of time to reduce pain levels and thus the need for air cooling in a bid to improve efficacy as well as patient tolerability. 

Monitoring PpIX fluorescence during real-time PDT in a reliable, quantitative manner is not trivial and to make highly accurate PpIX measurements, corrections for variations in tissue optical properties need to be made [24,25]. This has been achieved within skin cancer models [26] and human tissues [27,28]. Such corrections were not undertaken here as our purpose was to seek potential predictors of clinical outcome at the time of treatment in a relative manner within a considerably sized, pre-existing data set. Furthermore, wide variations in inter-lesional PpIX levels mean that measures of pre-treatment PpIX are less useful for determining clinical outcome at the time of light delivery than measuring diffuse PpIX changes during the first irradiation period. As considerable variations in PpIX levels can occur within an individual lesion [16], utilizing a standardized operating procedure to record PpIX fluorescence [16] is essential. Smaller lesions (<50 mm) have also been found to be more likely to exhibit homogeneous PpIX distribution than larger lesions [19]. PpIX fluorescence measurements have been reported here in arbitrary units, however we have previously calculated that mean estimates of PpIX levels of ~0.80 μM pre MAL application, ~10.00 μM post MAL application and ~0.75 μM post irradiation occurred in an individual skin lesion monitored with this fluorescence imaging system, with a good degree of accuracy being observed in the 0–10 μM range [16]. It has also been speculated that differences between the PpIX levels observed on the head/neck and lower leg [29] may be due to the temperature differences that exist at different body sites, as PpIX accumulation has been observed to occur faster at higher temperatures [30]. PpIX photobleaching has also previously been monitored clinically during sBCC ALA-PDT at a variety of different fluence rates [31]. In vivo studies have also indicated a positive correlation between PpIX photobleaching and cellular damage [32,33]. PpIX photobleaching particularly during the first minute of irradiation, is also strongly correlated to oxygen consumption during dermatological MAL-PDT [34]. No intervention that photobleaches the photosensitizer is suggested, simply observation of PpIX photobleaching as a surrogate for the photodynamic reaction in progress (as the production of singlet oxygen by MAL-PDT interacts with PpIX to produce a non PDT active photoproduct).

## 4. Materials and Methods 

### 4.1. Dermatological MAL-PDT

Patients attending Royal Cornwall Hospital for routine MAL-PDT for licensed indications (AK, BD and sBCC) were recruited following giving consent and with ethical approval from the Cornwall and Plymouth Research Ethics Committee. All subjects gave their informed consent for inclusion before they participated in the study. The study was conducted in accordance with the Declaration of Helsinki, and the protocol was approved by the Cornwall and Plymouth Research Ethics Committee (23/Q210/122). Patients were treated in accordance to National Institute of Health and Care Excellence guidelines [18] as part of a standard nurse-led dermatological PDT clinic. Patient age, gender, lesion type and exact lesion location were recorded. Participants had Fitzpatrick skin type I, II or III.

Any lesion crust was gently removed via curettage. MAL (Metvix^®^, 160 mg/g MAL, Galderma, Watford, UK) was then applied (~1 mm thick with 5 mm normal border) and an occlusive dressing applied. Three hours later, any excess MAL was wiped away and the lesion irradiated (Aktilite, Galderma, Watford, UK, 635 ± 5 nm, 37 Jcm^−2^, 70–100 mWcm^−2^) taking care to use the center part of the light array to irradiate the entire lesion plus a margin of normal surrounding skin utilizing the Aktilite spacer bar provided. Any use of pain relief was noted. After treatment, the lesion was covered with an occlusive dressing for 24 h. All lesions included in this study excepting the most superficial AK lesions [3] received two identical MAL-PDT treatments nine days apart in accordance with the routine PDT clinic schedule. This clinical decision was made by the Consultant Dermatologist who made the patient’s management plan indicating treatment with PDT in accordance with the NICE Guidelines [18].

A Consultant Dermatologist (blinded observer unaware of imaging results) clinically assessed all treatment areas at three months. Lesions were considered to have achieved complete clinical clearance if no visual evidence of the tumor remained. 

### 4.2. Fluorescence Imaging

A commercially available, validated [16], non-invasive fluorescence imaging system (Dyaderm, Biocam, Regensburg, Germany) [17] was employed to image each lesion prior to MAL application, immediately before irradiation and immediately after irradiation. This permitted PpIX accumulation and subsequent photobleaching during irradiation to be monitored. 

The fluorescence imaging system simultaneously collected and processed in real-time a normal colored image (from white light) and a PpIX fluorescence image (from blue light; 370–440 nm) using a filtered Xenon flash light source, a charged couple device camera and custom-made software (Dyaderm Pro v2, Biocam, Regensburg, Germany). Natural green spectrum autofluorescence was also imaged and subtracted from the image produced to ensure that the sole changes recorded resulted from PpIX. A synthetic PpIX fluorescence standard (Biocam, Regensburg, Germany) was also imaged on each clinic day to ensure system continuity and reproducibility. 

This process followed the standard protocol derived previously to enable reproducible images to be acquired and thus PpIX levels to be semi-quantified in a reliable manner with a piece of equipment originally designed for PDD [16]. This included a standardized warm up phase, consistent imaging distance/perpendicular angle, consistent light conditions (door shut/lights on), central orientation of the lesion and maximal camera activation of sixty seconds.

### 4.3. Data Analysis

Bitmap image exports were analyzed in NIH ImageJ software (http://rsb.info.nih.gov/ij/) using the same point within the lesion to record the mean greyscale values at each time point. Following data integrity checks, where the data held in Microsoft Excel was cross referenced with the original data in its entirety by one researcher and additional spot checks undertaken at 10% intervals by a second researcher, the entire data set was imported into STATA 14.1 for analysis in this non-interventional, observational study. 

This data set originally contained 270 entries for licensed lesions that had undergone fluorescence imaging during standard MAL-PDT. Any lesion that did not have complete three time point fluorescence data sets recorded for both MAL-PDT treatment cycles was removed. STATA’s drop duplicate command then selected at random, without bias, one data set from each participant where treatments/fluorescence imaging had been conducted on more than one lesion. This left 207 complete entries. Finally, any lesion where the clinical response at three months was not known was removed, producing 172 data sets for analysis. 

Lesion location was classified as head, body, arms, legs and acral. A further peripheral category was created, where acral sites were merged with lesions on the arms and legs. 

PpIX accumulation was calculated by subtracting pre-treatment fluorescence (before MAL application) from pre-irradiation fluorescence. PpIX photobleaching was calculated by subtracting post-irradiation fluorescence from pre-irradiation fluorescence. Comparisons between the three time points were initially made using paired *t*-tests and repeated measures ANOVA. 

Linear regression analyses were then utilized to analyze the difference in PpIX accumulation/photobleaching for a range of predictors including lesion type, lesion location, pain relief, age and gender. Logistic regression analysis was used to explore the odds of complete clinical clearance for a range of predictors including lesion location, lesion type, age, sex, use of ACD and PpIX accumulation/photobleaching. To investigate the potential of PpIX accumulation/photobleaching to predict clinical outcome ROC curve analyses were performed using the STATA roccomp command. All models were adjusted for age and sex.

## 5. Conclusions

Non-invasive PpIX fluorescence monitoring is therefore concluded to be a useful technique for increasing understanding of the mechanism of action of dermatological MAL-PDT. Both PpIX accumulation and photobleaching are important indicators of treatment success and anything that adversely affects them has the potential to reduce efficacy, particularly as PpIX photobleaching during the first irradiation period has been found to be an excellent predictor of clinical outcome. 

## Figures and Tables

**Table 1:** Demographic comparison of the participants with complete PpIX fluorescence data sets either with or without clinical outcome data available.

Demographic	All Patients Included	Patients with Clinical Outcome Data	* P Comparison
N	207	172	NA
Mean age (SD)	72.7 (10.2)	73.1 (10.4)	0.2
Male sex, N (%)	99 (47.8)	81 (47.1)	0.6
Lesion type:			
AK (%)	82 (39.6)	64 (37.2)	0.1
sBCC (%)	58 (28.0)	47 (27.3)	
BD (%)	67 (32.4)	61 (35.5)	
Lesion location:			0.9
Acral (%)	17 (8.2)	15 (8.7)	
Arms (%)	11 (5.3)	8 (4.7)	
Body (%)	47 (22.7)	39 (22.7)	
Head (%)	81 (39.1)	68 (39.5)	
Legs (%)	51 (24.6)	42 (24.4)	
Mean PpIX accumulation in arbitrary units (SD)	69.3 (32.1)	69.9 (31.7)	0.6
Mean PpIX photobleaching in arbitrary units (SD)	62.1 (32.2)	61.5 (30.2)	0.6
Pain relief used, N (%)	90 (43.5)	77 (44.8)	0.4

* P comparison calculated using age and sex adjusted logistic regression. SD: standard deviation, AK: actinic keratosis, sBCC: superficial basal cell carcinoma, BD: Bowen’s disease.

## References

[1] Taylor E.L., Brown S.B. (2002). The advantages of aminolevulinic acid photodynamic therapy in dermatology. J. Dermatol. Treat..

[2] Steinbauer J.M., Schreml S., Kohl E.A., Karrer S., Landthaler M., Szeimies R.M. (2010). Photodynamic therapy in dermatology. JDDG J. Dtsch. Dermatol. Ges..

[3] Morton C., Szeimies R., Sidoroff A., Braathen L. (2013). European Guidelines for Topical PDT. JEADV.

[4] Babilas P., Schreml S., Landthaler M., Szeimies R.M. (2010). Photodynamic therapy in dermatology: State-of-the-art. Photodermatol. Photoimmunol. Photomed..

[5] Bown S.G. (2012). How mainstream medicine sees photodynamic therapy in the United Kingdom. J. Natl. Compr. Cancer Netw..

[6] Fayter D., Corbett M., Heirs M., Fox D., Eastwood A. (2010). A systematic review of photodynamic therapy in the treatment of pre-cancerous skin conditions, Barrett’s oesphagus and cancers of the biliary tract, brain, head and neck, lung, oesophagus and skin. Health Technol. Assess..

[7] Peng Q., Berg K., Moan J., Kongshaug M., Nesland J. (1997). 5-Aminolevulinic acid-based photodynamic therapy: Principles and experimental research. Photochem. Photobiol..

[8] Scott M.A., Hopper C., Sahota A., Springett R., McIlroy B.W., Bown S.G., MacRobert A.J. (2000). Fluorescence Photodiagnosis and Photobleaching Studies of Cancerous Lesions using Ratio Imaging and Spectroscopic Techniques. Lasers Med. Sci..

[9] Wennberg A.M., Gudmundson F., Stenquist B., Ternesten A., Molne L., Rosen A., Larkö O. (1999). In vivo detection of basal cell carcinoma using imaging spectroscopy. Acta Dermato-Venereol..

[10] Ackermann G., Abels C., Karrer S., Baumler W., Landthaler M., Szeimies R.M. (2000). Fluorescence-assisted biopsy of basal cell carcinomas. Hautarzt.

[11] Siewecke C., Szeimies R.M. (2004). PDT and fluorescence diagnosis in dermatology. Hospital Pharmacy Europe.

[12] Bogaards A., Sterenborg H.J., Trachtenberg J., Wilson B.C., Lilge L. (2007). In vivo quantification of fluorescent molecular markers in real-time by ratio imaging for diagnostic screening and image-guided surgery. Lasers Surg. Med..

[13] Smits T., Kleinpenning M.M., Blokx W.A., van de Kerkhof P.C., van Erp P.E., Gerritsen M.J. (2007). Fluorescence diagnosis in keratinocytic intraepidermal neoplasias. J. Am. Acad. Dermatol..

[14] Hua Z., Gibson S.L., Foster T.H., Hilf R. (1995). Effectiveness of delta-aminolevulinic acid-induced protoporphyrin as a photosensitiser for photodynamic therapy in vivo. Cancer Res..

[15] Loh C.S., Vernon D., MacRobert A.J., Bedwell J., Bown S.G., Brown S.B. (1993). Endogenous porphyrin distribution induced by 5-aminolaevulinic acid in the tissue layers of the gastrointestinal tract. J. Photochem. Photobiol. B Biol..

[16] Tyrrell J., Campbell S., Curnow A. (2010). Validation of a non-invasive fluorescence imaging system to monitor dermatological PDT. Photodiagn. Photodyn. Ther..

[17] Jaap de L., van der Beek N., Neugebauer W.D., Bjerring P., Neumann H.A. (2009). Fluorescence detection and diagnosis of non-melanoma skin cancer at an early stage. Lasers Surg. Med..

[18] National Institute for Health and Care Excellence (NICE) (2006). Photodynamic therapy for non-melanoma skin tumours (including premalignant and primary non-metastatic skin lesions). NICE Interv. Proced. Guid..

[19] Tyrrell J., Campbell S., Curnow A. (2011). Monitoring the accumulation and dissipation of the photosensitiser protoporphyrin IX during standard dermatological methyl-aminolevulinate photodynamic therapy utilizing non-invasive fluorescence imaging and quantification. Photodiagn. Photodyn. Ther..

[20] Tyrrell J., Morton C., Campbell S., Curnow A. (2011). Comparison of PpIX accumulation and destruction during methyl-aminolevulinate photodynamic therapy (MAL-PDT) of skin tumours located at acral and non-acral sites. Br. J. Dermatol..

[21] Tyrrell J., Campbell S., Curnow A. (2010). The relationship between protoporphyrin IX photobleaching during real-time dermatological methyl-aminolevulinate photodynamic therapy (MAL-PDT) and subsequent clinical outcome. Lasers Surg. Med..

[22] Nissen C.V., Heerfordt I.M., Wiegell S.R., Mikkelsen C.S., Wulf H.C. (2017). Increased protoporphyrin IX accumulation does not improve the effect of photodynamic therapy for actinic keratosis: A randomized controlled trial. Br. J. Dermatol..

[23] Tyrrell J., Campbell S., Shore A., Curnow A. (2011). The effect of air cooling pain relief on protoporphyrin IX photobleaching during real-time dermatological methyl-aminolevulinate photodynamic therapy. J. Photochem. Photobiol. B Biol..

[24] Kim A., Khurana M., Moriyama Y., Wilson B. (2010). Quantification of in vivo fluorescence decoupled from the effects of tissue optical properties using fiber-optic spectroscopy measurements. J. Biomed. Opt..

[25] Middelburg T., Hoy C., Neumann H., Amelink A., Robinson D. (2015). Correction for tissue optical properties enables quantitative skin fluorescence measurements using multi-diameter single fiber reflectance spectroscopy. J. Dermatol. Sci..

[26] Sunar U., Rohrbach D.J., Morgan J., Zeitouni N., Henderson B.W. (2013). Quantification of PpIX concentration in basal cell carcinoma and squamous cell carcinoma models using spatial frequency domain imaging. Biomed. Opt. Express.

[27] Kanick S., Davis S., Zhao Y., Hasan T., Maytin E., Pogue B., Chapman M. (2014). Dual-channel red/blue fluorescence dosimetry with broadband reflectance spectroscopic correction measures protoporphyrin IX production during photodynamic therapy of actinic keratosis. J. Biomed. Opt..

[28] Rollakanti K., Kanick S., Davis S., Pogue B., Maytin E. (2013). Techniques for fluorescence detection of protoprophyrin IX in skin cancers associated with photodynamic therapy. Photonics Lasers Med..

[29] Kulyk O., Ibbotson S.H., Moseley H., Valentine R.M., Samuel I.D. (2015). Development of a handheld fluorescence imaging device to investigate the characteristics of protoporphyrin IX fluorescence in healthy and diseased skin. Photodiagn. Photodyn. Ther..

[30] Juzeniene A., Juzenas P., Kaalhus O., Iani V., Moan J. (2002). Temperature effect on accumulation of protoporphyrin IX after topical application of 5-aminolevulinic acid and its methyl ester and hexyl ester derivatives in normal mouse skin. Photochem. Photobiol..

[31] Cottrell W.J., Paquette A.D., Keymel K.R., Foster T.H., Oseroff A.R. (2008). Irradiance-dependent photobleaching and pain in delta-aminolevulinic acid-photodynamic therapy of superficial basal cell carcinomas. Clin. Cancer Res..

[32] Boere I.A., Robinson D.J., de Bruijn H.S., van den Boogert J., Tilanus H.W., Sterenborg H.J., de Bruin R.W.F. (2003). Monitoring in situ dosimetry and protoporphyrin IX fluorescence photobleaching in the normal rat esophagus during 5-aminolevulinic acid photodynamic therapy. Photochem. Photobiol..

[33] Kruijt B., de Bruijn H.S., van der Ploeg-van den Heuvel A., de Bruin R.W., Sterenborg H.J., Amelink A., Robinson D.J. (2008). Monitoring ALA-induced PpIX photodynamic therapy in the rat esophagus using fluorescence and reflectance spectroscopy. Photochem. Photobiol..

[34] Tyrrell J., Thorn C., Shore A., Campbell S., Curnow A. (2011). Oxygen saturation and perfusion changes during dermatological methyl-aminolevulinate photodynamic therapy. Br. J. Dermatol..

